# Dendrimeric Nanoarchitectures Mediated Transdermal and Oral Delivery of Bioactives

**DOI:** 10.4103/0250-474X.44589

**Published:** 2008

**Authors:** V. Gajbhiye, P. Vijayaraj Kumar, A. Sharma, A. Agarwal, A. Asthana, N. K. Jain

**Affiliations:** Pharmaceutics Research Laboratory, Department of Pharmaceutical Sciences, Dr. Hari Singh Gour University, Sagar-470 003, India

**Keywords:** PAMAM dendrimer, transdermal, oral, transcellular, paracellular

## Abstract

Transdermal route is an evolving panorama in novel drug deliverance and with oral route they proffer immense potential. Most recently there is hastening in approaches for delivering bioactives via these routes, amongst them revolution has been made by dendrimers. Encapsulation and conjugation of bioactives with these virus sized robots have shown immense employment for delivery of hydrophobic and labile remedies. Transport of these nano-cruises from corner to corner of skin and through epithelial hurdle of gastrointestinal tract depends upon dendrimer characteristics. An improved thoughtful of these characteristics is an obligation for their use in these rambling fields. These characteristics embrace generation size, molecular weight, surface charge, incubation time and concentration. This context demarcates the imperative role of dendrimers in transdermal and oral drug delivery. This review also highlights concerning mechanism of convey of nanoarrays via epithelial hurdle of GIT.

Dendrimers consist of a central core from which many arms emanate making it highly branched nano-structure with sky-scraping pharmaceutical and biomedical potentials including gene and oligonucleotide delivery^1^–^4^, vehicle for delivery of bioactives^5^–^8^, development of vaccine^9^, diagnostic field^10^, solubilization of hydrophobes^11^ and treating cancer with chemotherapeutics, photodynamic therapy and boron neutron capture therapy^12^–^16^. Moreover these nano-objects are shown to have their own pharmacological activity as nanobiomolecular-doctor against a variety of conditions like activity against HSV-1 (Herpes Simplex Virus)^17^ and against HIV (Human immunodeficiency virus)^18^, anticancer activity^19^,^20^ and antimicrobial activity^21^. As weighed against linear polymers, dendrimers provide reproducible pharmacokinetic properties. Stepwise synthesis of these nano-carriers leads to well-defined groups and controlled multivalency at their periphery. During stepwise synthesis a considerable change in conformation takes place escorting amend in constitution to globular form. The groups at periphery are constructive for attachment of numerous drug molecules^22^, targeting moieties^23^,^24^, solubilizing agents^25^, spacers^26^ and furthermore for interaction with biological membranes. In the middle of different dendrimers, polyamidoamine (PAMAM) are cationic in nature having surface amino groups, which have been used for transdermal and oral application, albeit few reports are also there on use of polylysine dendrimer for oral drug delivery. First testimonial in favor of PAMAM dendrimers were bequeathed via Tomalia *et al*^27^. Since then these biomolecular robots has been drawn on as a potential drug emancipation organization for genetic fabrics, anticancer agents, antiinflammatory medicines as well as in diagnostic applications^28^–^31^.

The studies have shown that dendrimer adopt paracellular^32^ and transcellular route^33^ for crossing the epithelial barrier of the cells. These uptake routes for dendrimers dish up as supplementary itinerary for accessing systemic circulation. Transport of these nano-projectiles across of skin and through epithelial barrier depends upon dendrimer characteristics. An improved thoughtful of these characteristics is an obligation for their use in these rambling fields. These characteristics embrace generation size^34^, molecular weight^35^, surface charge^36^, incubation time and concentration^37^. Efflux transport and surface modification on dendrimers also affect their internalization. In this framework the key explorations in potential of dendrimers for transdermal and oral drug deliverance and mechanism of crossing epithelial barrier have been highlighted.

## DENDRIMER MEDIATED TRANSDERMAL DRUG DELIVERY

In recent era, transdermal route has rival with oral route as the most flourishing groundbreaking investigation locale in the drug delivery. Skin intrinsic barrier furnish a foremost confront in transdermal drug deliverance. Stratum corneum, the outermost cover of the skin, offers principal hurdle for diffusion of hydrophilic ionizable bioactives. Novel epoch go forward in transdermal drug delivery system is the dendrimer mediated transdermal delivery of bioactives. The virus sized cruises have been lucratively used for transporting the bioactives from corner to corner of the skin.

**Fig. 1:**
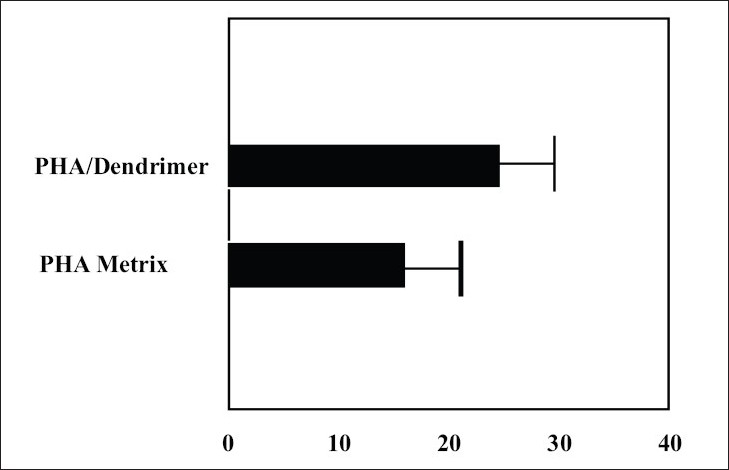
Tamsulosin permeation through shed snake skin. PHA containing dendrimer matrices showed higher permeation of tamsulosin through shed snake skin as compared to PHA matrix. [Adapted from Ref. 38 with slight modification]. Reproduced with permission from Elsevier.

The only nano-organized bullets, which have been used for transdermal delivery of bioactives, are PAMAM dendrimers. For mostly Wang *et al,*^38^ brought into focus PAMAM dendrimers in transdermal drug delivery system with the object to amplify the incursion of tamsulosin across the skin. They synthesized polyhydroxyalkanoate (PHA) matrix restraining PAMAM dendrimers. Observations point out penetrated quantity of tamsulosin through snake skin was 15.7 μg/cm^2^/d and 24μg/cm^2^/d from PHA and PAMAM dendrimers containing PHA matrices, correspondingly. These consequences illustrate that being there PAMAM dendrimers augment the diffusion of tamsulosin. They conjectured that the PAMAM dendrimers itself does not voyage in the interior of the skin; however, it takes steps as polymeric skin permeation enhancer by altering the macroscopic constitution of water in the solution. In the midst of automatic flow through diffusion cell apparatus, they scrutinized the pretreatment outcome of dendrimer on tamsulosin penetration using shed snake skin, which portray the knack of dendrimer as permeation booster^38^ (fig. 1).

They lugged out X-ray analysis of PHA matrix amid tamsulosin and dendrimer en route for shed light on the augmentation upshot by the nanostructures. It demonstrates Tamsulosin crystallized finely in PHA matrix in charisma of dendrimer as weighed against PHA matrix devoid of dendrimer wherein tamsulosin barely crystallized. Being there in PHA matrix, nanorobots persuade decidedly ordered orientation of tamsulosin crystals, which promoted drug release. Attributed to restricted dissemination direction, tamsulosin diffusion was facilitated through snake skin^39^.

**Fig. 2:**
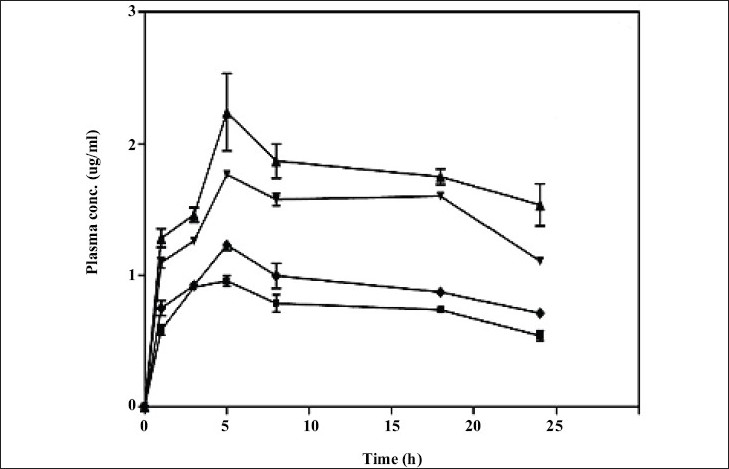
Plasma concentration achieved by different Indomethacin formulations. The NH2 terminated G4 PAMAM dendrimer (▴) showed maximum plasma concentration as compared to OH terminated G4 PAMAM dendrimer (▾), G4.5 PAMAM dendrimer (♦) and plain indomethacin (●) [Adapted from Ref. 40 with slight modification]. Reproduced with permission from Elsevier.

Chauhan *et al*^40^ furthermore accounted transdermal utilization of dendrimers. To enhance transdermal permeation of indomethacin by using PAMAM dendrimers was their aspiration. They conferred indomethacin transdermally by means of three dissimilar sorts of PAMAM dendrimers. Motive behind the selection of PAMAM dendrimers was solubility enhancement of hydrophobic drugs by these dendrimers, which grounds staging of drug in the direction of the biological coverings at home with added diffusible form. The sort of PAMAM dendrimers used were G4 containing surface amino moieties, G4.5 containing surface carboxylate moieties and G4 containing surface hydroxyl moieties^40^ (fig. 2).

Authors stay forward that dendrimer proceed like a hauler escorting drug partition into stratum corneum owing to lugging of drug glimmers towards the skin surface in more solubilized form. The studies designated that solubility enhancement of indomethacin was highest with G4 amino terminated dendrimer formulation. Amino terminated dendrimer furthermore augment flux to a privileged extent as put side by side to hydroxyl and carboxylate terminated dendrimer. Awfully trivial enhancement of flux revealed next to carboxylate terminated formulation. The pharmacokinetic studies illustrate that utmost drug concentration was through amino and hydroxyl terminated dendrimer formulation as contrast to pure drug. The area under curve as of 0 to 24 h of amino and hydroxyl terminated dendrimers were respectively, 2.27 and 1.95 folds greater than gratis drug suspension. Consequences of percentage diminution in rat maul edema volume as well illustrated higher affinity of reduction via amino and hydroxyl terminated dendrimer formulation, which was 1.6±0.15 and 1.5±0.13 folds correspondingly than the free drug. Awful subsidiary augment in pharmacokinetic and pharmacodynamic parameters was bringing into being in the case of carboxylate terminated dendrimer formulation^40^. In spite of transdermal route dendrimers have been utilized for delivering bioactives through conventional oral route also.

Recently Yiyun *et al*^41^ also investigated ability of PAMAM dendrimers for transdermal delivery of ketoprofen and diflunisal. PAMAM dendrimers have significantly enhanced the accumulated permeated amount of both drugs *in vitro* during permeation studies with excised rat skin, in 24 h compared with plain drugs. They also confirmed the ability by antinociceptive studies using the acetic acid induced writhing model in mice, in which the dendrimer complex have shown prolonged pharmacodynamic profile for both drugs after transdermal administration. The blood level studies of both drugs have demonstrated that bioavailability was 2.73 and 2.48 times higher for ketoprofen-PAMAM dendrimer complex and diflunisal-PAMAM dendrimer complex, respectively, as compared to pure drug suspensions. The authors suggested that PAMAM dendrimers can successfully facilitate skin diffusion of NSAIDs and this potential application of dendrimers can be employed for development of novel transdermal formulations.

## DENDRIMER MEDIATED ORAL DRUG DELIVERY

**Fig. 3:**
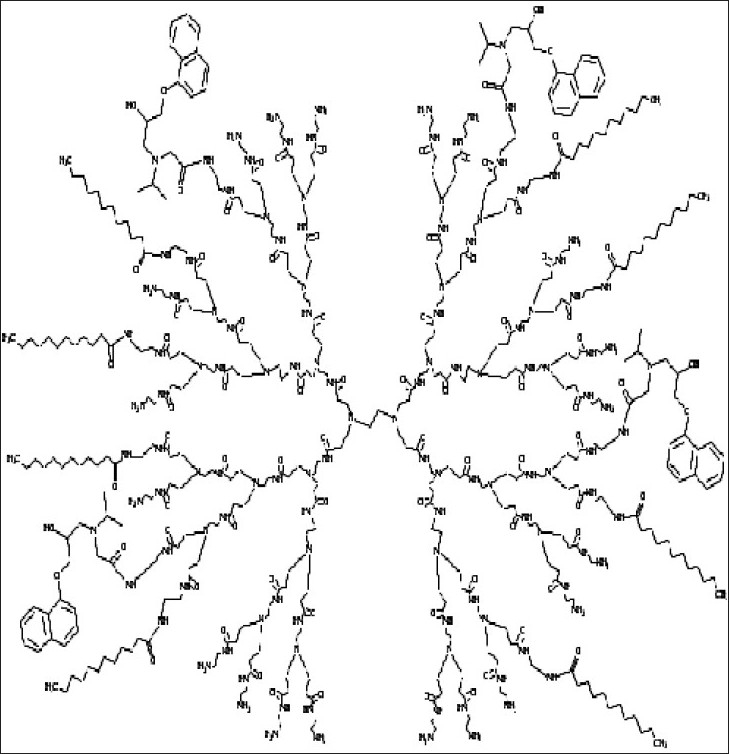
Structure of propranolol-lauroyl chain-PAMAM (G3) dendrimer.

Oral route has always been preferred over perenteral route due to snag, toxicity and non-patient compliance of perenteral route. Dendrimers also have been lucratively used for delivering hydrophobes because of their improved solubilization characteristics and labile bioactives for enhancing bioavailability via this route. D’Emanuele *et al*^42^ synthesized prodrug of propranolol by conjugating to G3 and lauroyl-G3 PAMAM dendrimers (fig. 3) with the endeavor to establish the transport of propranolol through human colon adenocarcinoma cell line (Caco-2 cell). Their aspiration was to amplify the solubility of propranolol and to evade P-glycoprotein (P-gp) transport mediated efflux of drug for which it is a recognized substrate. Their observation illustrates dwindle toxicity of lipid chain-drug-dendrimer conjugate over non-modified dendrimer on Caco-2 cells^42^ (Table 1).

**TABLE 1:** CYTOTOXICITY OF PAMAM DENDRIMER AND DENDRIMER CONJUGATES ON CACO-2 CELLS

Dendrimer	IC_50_ (mM)
G3	0.141±0.004
G3P2	0.155±0.005
G3P4	0.161±0.002
G3P6	0.164±0.001
G3L2P2	0.235±0.006
G3L6P2	0.492±0.003
G3L2P6	0.172±0.002

IC_50_ (mM) = Concentration for 50% inhibition of mitochondrial dehydrogenase activity. Mean ± SD, n=5. [Adapted from ref. 42]. Reproduced with permission from Elsevier

These consequences show that the apparent partition coefficient (P_app_) of propranolol from apical-to-basolateral (AB) direction was less than basolateral-to-apical (BA) direction due to P-gp efflux. In charisma of dendrimer its P_app_ was less as compared to charisma of cyclosporin A, which causes enhancement in P_app_ of propranolol, signifying that nano-objects do not accomplish as P-gp efflux inhibitor. Nevertheless, on covalent attachment of propranolol to G3 dendrimer P_app_ increased irrespective of the number of attached propranolol molecules, which further increased on lauroyl chains conjugation on dendrimers owing to upshot of lipid chains on endocytosis. The transepithelial electrical resistance (TEER) measurement designate that enhanced permeability through cell monolayer is owing to opening of tight junctions by lipid chain-drug-dendrimer conjugates^42^.

**Fig. 4:**
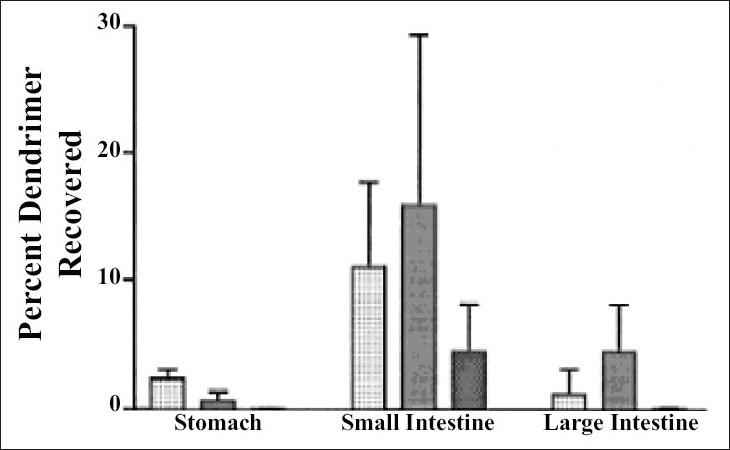
Dendrimer uptake from GIT of female Sprague Dawley rat. Light white, light black and dark black bars represent dendrimer uptake from GIT of female Sprague Dawley rat after 3, 6 and 24 h, respectively. The percentage of dendrimer recovered from small intestine was found highest. [Adapted from Ref. 43]. Reproduced with permission from Elsevier.

Florence *et al*^43^ primed lysine dendrimers covering 16 alkyl (C_12_) chains at the periphery. Their intention was to study absorption of these dendrimer from Sprague-Dawley rats following oral administration for which they preferred G4 dendrimer with a diameter of 2.5 nm having molecular weight of 6300 and log P (octanol/water) of 1.24. The peptide dendrimer they selected have superior solubilizing capability for hydrophobic drugs, which leads to improved absorption. For assessing uptake in blood, liver, kidney, stomach, intestine, spleen they provided a dose of 14 mg/kg orally to the fasted rats and for assessing uptake through Payer’s patches and enterocytes they orally administered a dose of 28 mg/kg. The percentage of dendrimer recovered from small intestine, large intestine and blood was 15, 5 and 3%, respectively after 6 h of oral dose (fig. 4). Only 1.5, 0.1 and 0.5% were found respectively in liver, spleen and kidney. 1 and 4% was absorbed by the Payer’s patches and enterocytes of small intestine, respectively after 3h; while 0.3 and 4% dendrimer was absorbed in Payer’s patches and enterocytes of large intestine, respectively after 12 h. The total percentage of dendrimer absorbed was greater for Payer’s patches than enterocytes for small intestine as far as target tissue weight is concerned, while opposite was established for large intestine. Authors compared absorption results of dendrimers with polystyrene particles having size ranging from 50-3000 nm, which was less for dendrimers as weighed against polystyrene particles. They concluded that aggregation of dendrimer and partial dendrimer form supramolecular assemblies, which was responsible for the lower uptake and absorption of dendrimer was not greater than that of 50 nm latex particle^43^,^44^.

**Fig. 5:**
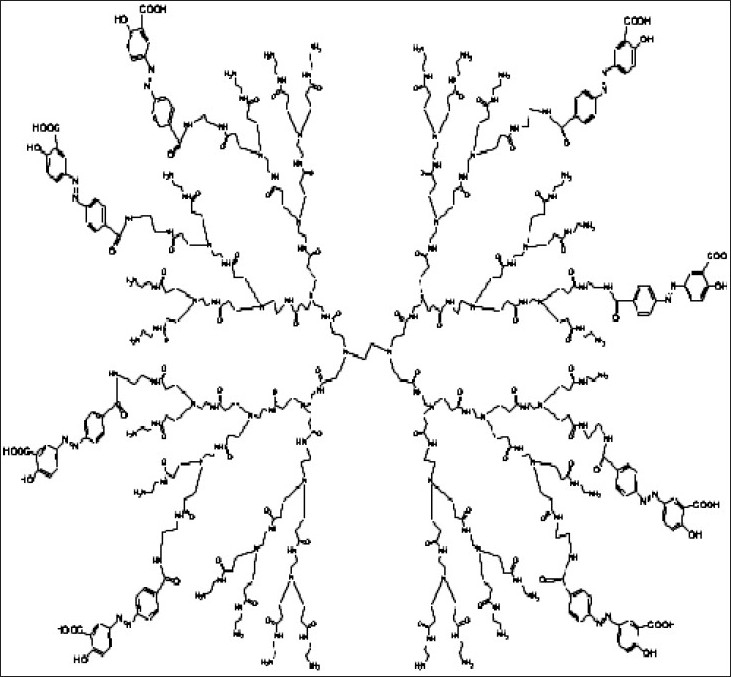
Structure of PAMAM (G4)-PABA-SA Conjugate.

Furthermore, Wiwattanapatapee *et al*^45^ evaluated the potential of PAMAM dendrimer for colonic delivery of 5-aminoslicylic acid by conjugating the drug to the nano-robots with the help of two dissimilar spacers, which are p-aminobenzoic acid (PABA) (fig. 5) and p-aminohippuric acid (PAH). Authors compared the activity of these conjugates with PABA-salicylic acid (PABA-SA) and PAH-SA conjugates. Their result give us an idea that the PAH as a spacer craft the dendrimer proficient for carrying the drug, about three times more than the PABA as spacer. This outcome is due to less steric hindrance of –COOH group created by PAH in contrast to PABA. For study in stomach homogenate they selected isotonic acetate buffer (pH 4.5) and phosphate buffer saline (pH 6.8) for small intestine and cecal homogenate of rat. After 12 h, 23 and 38% of dose released in the form of 5-ASA from dendrimer-PABA and dendrimer-PAH conjugate, respectively in cecal homogenate of the rat, which was increased to 45 and 57% after 24 h, respectively, while in small intestine the percentage of 5-ASA released was 4.5-7.2% from both the conjugates after 12 h. The drug release was found to be higher in the case of both dendrimer conjugates as weighed against PABA-SA and PHA-SA conjugates. Authors suggested that the drug release from the dendrimer conjugate was due to the splitting of amide bond formed between dendrimer and spacer and azo bond formed between spacer and SA. The amplified release from nano-conjugates in colonic homogenate is principally due to cleavage of azo bond by azoreductase enzyme in colon^45^. Na *et al*^46^ investigated oral delivery ketoprofen-PAMAM dendrimer complex. Acetic acid induced writhing model in mice have shown prolonged pharmacodynamic effect of the ketoprofen-PAMAM dendrimer complex. The blood level studies of above complex also conclude that PAMAM dendrimer shows sustained release of ketoprofen. For effective delivery of drugs through oral route, the mechanism of transport of dendrimers across the intestinal epithelium should be clearly understood.

### Transport mechanism of dendrimer through epithelial barrier:

For delivering bioactives in systemic circulation nanoprojectiles should cross the biological barrier effectively. The penetration of dendrimer across epithelial barrier depends upon many factors including generation size, molecular weight, surface charge, efflux transport and surface modification.

### Effect of generation size and molecular weight:

Tajarobi *et al*^35^ inspected permeability of FITC labelled PAMAM (G0-G4) dendrimers through Madin-Darby Canine Kidney (MDCK) cell lines (Table 2), which has same permeability characteristics for passively transported compounds as that of Caco-2 cell lines. At 37° the permeability of dendrimer through MDCK cell lines were observed in AB direction. Amongst different dendrimers G4 dendrimers was found to have highest permeability while G2 dendrimers had lowest permeability (Table 2). They established that the higher permeability of G4 is due to higher interaction with cell surface. The integrity of cells was examined by observing permeability of mannitol in the presence and absence of dendrimers. They found nine fold increase in permeability of mannitol in presence of dendrimers as compared with absence of dendrimers. These results indicate transepithelial transport of the dendrimers through MDCK cell lines. According to authors the transport of dendrimer through MDCK cell lines depends on size of the dendrimers and their interaction with cell surface^35^.

**TABLE 2:** PARTITION COEFFICIENT FOR DIFFERENT MOLECULAR WEIGHT PAMAM DENDRIMERS ACROSS MDCK CELL

Generation	Molecular weight (Da)^a^	Diameter (Å)^a^	No. of surface amino groups per molecule^a^	P_app_×10^6^ (cm/s)^b^
G0	517	15	4	1.02 (±0.084)
G1	1430	22	8	1.08 (±0.064)
G2	3256	29	16	0.076 (±0.0056)
G3	6909	36	32	0.55 (±0.031)
G4	14215	45	64	16.1 (±0.58)

a Reported by Tomalia *et al*^47^.

b Permeability is the mean ± SEM of three analyzed MDCK monolayers. [Adapted from Ref. 35]. Reproduced with permission from Elsevier

Hong *et al*^34^ used atomic force microscopy, enzyme assays, flow cell cytometry and fluorescence microscopy techniques for investigating interaction and hole formation by the PAMAM (G7) dendrimers in lipid bilayers, KB and Rat2 cell membranes. They observed cytotoxicity of amine terminated G7, G5 and acetamide terminated G5 PAMAM dendrimers by release of cytosolic enzymes luciferase (Luc) and Lactase dehydrogenase (LDH) from KB and Rat2 cells. Cytotoxicity was found to be concentration dependent at 37°, as G5-NH_2_ dendrimer concentration was increased, cytotoxicity also increased, while no release of LDH was found in case of G5-Ac dendrimers from both cell types. However, at 6° no release of LDH was found from G5-NH_2_ and G5-Ac dendrimer in both cell types. Their results illustrate the generation size of the dendrimer effect cells too because LDH release was more in case of G7-NH_2_ dendrimer at 37 and 6° as compared to G5-NH_2_ dendrimer, which is proficient of causing LDH release at 37° and not at 6°. Liberation of cytosolic enzymes LDH and Luc in the charisma of dendrimers designate increased permeability of cells owing to hole formation. They have established that membrane permeability again became normal after removal of dendrimers and dendrimer can cross the membrane by receptor-mediated endocytosis^34^.

Wiwattanapatapee *et al*^32^ examined the effect of size of dendrimer on uptake and convey through rat intestine *in vitro* by everted rat intestine sac model. They took cationoic PAMAM dendrimers of G3 and G4 and anionic PAMAM dendrimers of G2.5, G3.5, G5.5 having single amino group, which was radioiodinated. The rate of tissue uptake of radioiodinated anionic PAMAM dendrimer of G5.5 had significantly higher value with endocytic indices (EI) equal to 2.48±0.51 μl/mg protein/h than the uptake of radioiodinated G2.5 and G3.5 PAMAM dendrimers having values EI equal to 0.6-0.7 μl/mg protein/h. On the other hand the serosal transfer rate of all radioiodinated PAMAM dendrimers were analogous with EI in a range of 3.4-4.4 μl/mg protein/h, which was about 70-80% of the total radioactivity^32^.

### Effect of surface charge:

Tajarobi *et al*^35^ established that the higher permeability of G4 PAMAM dendrimers amongst G0-G4 was due to high positive charge, which interact with negative cell surface. Lower generations than G4 have less positive charge for interaction with the cell surface^35^. El-Sayed *et al.*^36^ investigated the effect of number of surface amino groups on the permeability of PAMAM dendrimer across Caco-2 cell monolayers. They confirmed permeability of FITC-PAMAM (G0-G4) dendrimer in both AB and BA directions. The integrity and viability of the cells was investigated by measuring TEER and LDH release, respectively. There was a decrease in TEER, which was concentration and generation dependent. The LDH release studies explain that G0-G2 were non-toxic to the cells as compared to higher generations. In presence and absence of PAMAM dendrimers, the permeability of ^14^C-mannitol was also observed in both AB and BA directions at pH 7 and temperature 37°, which was found to increase with generation size. The permeability of ^14^C-mannitol was found to be higher in BA direction as compared to AB direction owing to some efflux transporters and also due to presence of tight junctional proteins in apical membrane and not in basolateral membranes. Their results confirm increased interaction of dendrimer with cells as generation increases, as permeability of ^14^C-mannitol was higher in presence of higher generations of dendrimers. Authors proposed that the permeability of dendrimer was due to interaction of positively charged dendrimers with negatively charged epithelium. This is the reason behind opening of tight junctions and their transport by paracellular route^36^.

### Effect of incubation time:

El-Sayed *et al*^37^ have investigated mechanism of convey of PAMAM dendrimers (G2) all the way through Caco-2 cell monolayers. They examined involvement of adsorptive endocytosis and P-gp efflux system in transport of dendrimers athwart the cells. The AB permeability of G2 PAMAM dendrimers were examined at 37 and 4°. They found that permeability was augmented with amplified incubation time observed at 90 and 150 min at 37°, while it was less at 4° than 37° at both incubation times. It implies adsorptive endocytosis contribution in transport, which is also affected by the temperature conditions^37^.

### Effect of concentration:

Tajarobi *et al*^35^ have shown that the P_app_ of G2 PAMAM dendrimers depends upon concentration of dendrimers, which was found to be highest at 200-300 μg/ml, about three times from that of 130-200 μg/ml. The partition coefficient was found to be similar for 50 μg/ml as that of 100 μg/ml but lower than 130-200 μg/ml^35^.

**Fig. 6:**
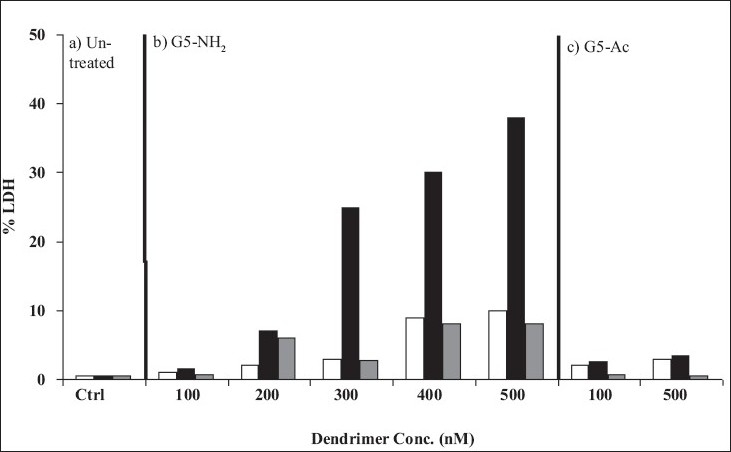
Enzyme release of Rat incubated with (a) G5-NH_2_ and (b) G5-Ac. White and dark black bars represent the release after 1h and 3 h incubation with dendrimer solutions, respectively. Light black bars represent enzyme release after 1 h incubation with dendrimers followed by washing and 2 h incubation in pure PBS buffer to allow recovery of membrane integrity. Reprinted with permission from [34] with slight modification. Copyright (2004) American Chemical Society.

Hong *et al*^34^ conducted fluorescence microscopy of PAMAM dendrimer at 37° and found significant internalization of FITC-G5-NH_2_ dendrimers at concentration of 200 nM in Rat2 cells, which was not considerable at 6°. They established concentration dependent release of LDH and Luc enzyme secretion from the cells in existence of dendrimers^34^ (fig. 6).

Wiwattanapatapee *et al*^32^ found that as compared to anionic PAMAM dendrimers, cationic dendrimers of G3 and G4 do have elevated tissue uptake value (EI = 3.3-4.8 μl/mg protein/h) than the serosal transfer (EI = 2.3-2.7 μl/mg protein/h) for first 60 minutes. According to authors, rate of uptake of cationic dendrimers improved with increasing concentration, while this was not the case with anionic dendrimers excepting G5.5. They notified that mechanism of uptake was opening of intercellular junction fleetingly and endocytosis. They suggested that the dendrimer possibly will also have used the paracellular pathway^32^.

### Effect of efflux transporters:

El-Sayed *et al*^37^ also investigated effect of P-gp efflux system on dendrimer transport, for which AB and BA permeability of ^14^C-paclitaxel in the presence of G2 PAMAM dendrimers was monitered. They concluded that the permeability of G2 PAMAM dendrimer across Caco-2 cell monolayers is affected by the combination of adsorptive endocytosis and paracellular transport. They found that permeability of ^14^C-paclitaxel was higher in BA direction as compared to AB direction but permeability of dendrimers was same in both directions, suggesting that the P-gp efflux system does not affect dendrimer transport. They also informed that transporters other than P-gp efflux system can result in efflux of dendrimer. So completely omitting the effect of efflux system on dendrimer transport is not possible. The permeability of G2 PAMAM dendrimers and ^14^C-mannitol (known paracellular permeability marker) across the cells increased in the presence of palmitoyl carnitine, which is a paracellular route transporter. These results designate that paracellular route also contributes in the transport of the dendrimer across the cells. These results were supported by decrease in TEER and amplified permeability of ^14^C-mannitol in the presence of G2 PAMAM dendrimers through Caco-2 cell monolayers^37^.

### Effect of surface modification:

Jevprasesphant *et al*^48^ investigated mechanism of transport of PAMAM and surface modified PAMAM dendrimers through Caco-2 cell monolayers. Authors found that the transport of dendrimers and dendrimer conjugates could occur through both paracellular pathway and endocytosis-mediated transepithelial transport through transcellular route. They opted for cationic PAMAM dendrimers of G3, G4, G5 and anionic PAMAM dendrimers of G2.5 and G3.5. By measuring P_app_ of ^14^C-mannitol in both directions and in presence or absence of dendrimer and lauroyl conjugated dendrimer, they examined effect of dendrimer on integrity of Caco-2 cell monolayers. They found improved P_app_ of ^14^C-mannitol in the presence of simple dendrimers, which was more pronounced in the presence of lauroyl conjugated dendrimers due to permeation augmenting effect of lipid chains. The P_app_ was found to be more in BA direction than AB direction. They also observed effect of EDTA and colchicine on permeability of simple and lauroyl conjugated dendrimers. The EDTA, which opens the tight junction, was used to investigate contribution of paracellular pathway in dendrimer transport; and colchicine, an endocytosis inhibitor, was used to investigate contribution of transepithelial pathway in dendrimer transport across the cells. The above results specify paracellular and transcellular transport of dendrimer and dendrimer conjugates^48^.

Jevprasesphant *et al*^33^ also investigated transport of PAMAM dendrimers and surface modified PAMAM dendrimers with lauroyl chain through Caco-2 cell monolayers by flow-cytometry, scanning microscopy and transmission electron microscopy. Their results have divulged that the endocytosis played a major role in transepithelial transportation of PAMAM dendrimers from side to side of the cells. For flow cytometry study FITC-dendrimer conjugate was incubated with Caco-2 cell monolayers. For distinguishing dendrimer internalized and dendrimer remained on the cell surface, trypan blue was used as quencher of FITC florescence. Flow cytometry results disclosed that florescence was measured more inside the cells in the case of FITC-dendrimer conjugate, which was still higher for FITC-lauroyl chain-dendrimer conjugate indicating the lauroyl chains had increased the internalization of dendrimers. For confocal laser scanning microscopy Caco-2 cell monolayers were incubated with FITC-PAMAM dendrimer and FITC-lauroyl chain-PAMAM dendrimer. Scanning microscopy revealed that internalization through cells was unswerving for both FITC-PAMAM dendrimer and FITC-lauroyl chain-dendrimer conjugates. For transmission electron microscopy they prepared gold-labelled PAMAM dendrimers for locating the position of dendrimer inside the cells. These gold-nano conjugates were then incubated with the Caco-2 cell monolayers. Later ultra thin sections of these cells were observed with transmission electron microscopy. The TEM shows attachment of dendrimer to the surface of cells, internalization and localization into endosomes^33^.

## CONCLUSION

Pulled off ensues from above explorations craft our psyche that the dendrimer can be proficiently utilized in transdermal and oral drug delivery system with immense accomplishment. Considering the patient compliance, oral and transdermal delivery remains a preferred option as weighed against perenteral route. Oral and transdermal immunization by proficient vaccine can be interesting future prospect of dendrimers. Most of the bioactives are labile and hydrophobic, which can be made more bioavailable by transdermal and oral routes, as delivered by dendrimers. Studies that can enumerate the paracellular and transcellular transport and nature of the interaction with epithelial cells of these nano-organized bullets are still deficient. At higher concentrations and incubation times dendrimers have shown toxicity, requiring more biocompatible dendrimers for these applications. The factors discussed above and mechanism of transport of dendrimer should be optimized for specific delivery of bioactives. Effect of efflux transporters and dendrimer localization is still a mysterious part. Nevertheless merely few canvassers have assessed this potential and it requires more investigation in this meadow. Ensues conversed above should unravel new horizon in transdermal and oral utilization of nano-projectiles for pollster of the planet.

## References

[1] Hudde T, Rayner SA, Comer RM, Weber M, Isaacs JD, Waldmann H (1999). Activated polyamidoamine dendrimers: A nonviral vector for gene transfer to the corneal endothelium. Gene Ther.

[2] Zinselmeyer BH, Mackay SP, Schatzlein AG, Uchegbu IF (2002). The lower generation polypropyleneimine dendrimers are effective gene transfer agents. Pharm Res.

[3] Yoo H, Juliano RL (2000). Enhanced delivery of antisense oligonucleotides with fluorophore-conjugated PAMAM dendrimers. Nucl Acids Res.

[4] Yoo H, Sazani P, Juliano RL (1999). PAMAM dendrimers as delivery agents for antisense oligonucleotides. Pharm Res.

[5] Bhadra D, Bhadra S, Jain S, Jain NK (2003). A PEGylated dendritic nanaparticulate carrier of β uorouracil. Int J Pharm.

[6] Khopade AJ, Caruso F, Tripathi P, Nagaich S, Jain NK (2002). Effect of dendrimer on entrapment and release of bioactive from liposomes. Int J Pharm.

[7] Bhadra D, Bhadra S, Jain NK (2005). PEGylated peptide-based dendritic nanoparticulate systems for delivery of artemether. J Drug Del Sci Tech.

[8] Bhadra D, Bhadra S, Jain NK (2006). PEGylated peptide dendrimeric carriers for the delivery of antimalarial drug chloroquine phosphate. Pharm Res.

[9] Cruz LJ, Iguilar JC, Gonzalez LJ, Reyes O, Albericio F, Andreu D (2004). A comparative study of different presentation strategies for an HIV peptide immunogen. Bioconjug Chem.

[10] Wiener EC, Brechbiel MW, Brothers H, Magin RL, Gansow OA, Tomalia DA (1994). Dendrimer-based metal chelates: A new class of magnetic resonance imaging contrast agents. Magn Reson Med.

[11] Chauhan AS, Jain NK, Diwan PV, Khopade AJ (2004). Solubility enhancement of indomethacin with poly(amidoamine) dendrimers and targeting to inflammatory regions of arthritic rats. J Drug Target.

[12] Majoros IJ, Myc A, Thomas T, Mehta CB, Baker JR (2006). PAMAM dendrimer based multifunctional conjugate for cancer therapy: Synthesis, characterization and Functionality. Biomacromolecules.

[13] Wu G, Barth RF, Yang W, Kawabata S, Zhang L, Green-Church K (2006). Targeted delivery of methotrexate to epidermal growth factor receptor-positive brain tumors by means of cetuximab (IMC-C225) dendrimer bioconjugates. Mol Cancer Ther.

[14] Battah SH, Chee CE, Nakanishi H, Gerscher S, MacRobert AJ, Edwards C (2001). Synthesis and biological studies of 5-aminolevulinic acid-containing dendrimers for photodynamic therapy. Bioconjug Chem.

[15] Battah S, O’Neill S, Edwards C, Balaratnam S, Dobbin P, MacRobert AJ (2006). Enhanced porphyrin accumulation using dendritic derivatives of 5-aminolaevulinic acid for photodynamic therapy: an *in vitro* study. Int J Biochem Cell Biol.

[16] Barth RF, Adams DM, Soloway AH, Alam F, Darby MV (1994). Boronated starburst dendrimer-monoclonal antibody immunoconjugates: Evaluation as a potential delivery system for neutron capture therapy. Bioconjug Chem.

[17] Bourne N, Stanberry LR, Kern ER, Holan G, Matthews B, Bernstein DI (2000). Dendrimers, a New Class of Candidate Topical Microbicides with Activity against Herpes Simplex Virus Infection. Antimicrob Agents Chemother.

[18] Witvrouw M, Fkkert V, Pluymers W, Matthews B, Mardel K, Schls D (2000). Polyanionic (i.e., polysulfonate) dendrimers can inhibit the replication of human immunodeficiency virus by interfering with both virus adsorption and later steps (reverse transcriptase/integrase) in the virus replicative cycle. Mol Pharmacol.

[19] Vannucci L, Fiserova A, Sadalapure K, Lindhorst TK, Kuldova M, Rossmann P (2003). Effects of N-acetyl-glucosamine-coated glycodendrimers as biological modulators in the B16F10 melanoma model *in vivo*. Int J Oncol.

[20] Dufes C, Keith WN, Bilsland A, Proutski I, Uchegbu IF, Schatzlein AG (2005). Synthetic anticancer gene medicine exploits intrinsic antitumor activity of cationic vector to cure established tumors. Cancer Res.

[21] Chen CZ, Beck-Tan NC, Dhurjati P, Van Dyk TK, LaRossa RA, Cooper SL (2000). Quaternary ammonium functionalized poly(propylene imine) dendrimers as effective antimicrobials: Structure-activity studies. Biomacromolecules.

[22] Yang H, Lopina ST (2003). Penicillin V-conjugated PEG-PAMAM star polymers. J Biomater Sci Polymer Edn.

[23] Qualmann B, Kessels MM, Musiol HJ, Sierralta WD, Jungblut PW, Moroder L (1996). Synthesis of boron rich lysine dendrimer as protein labels in electron microscopy. Angew Chem Int Ed Engl.

[24] Patri AK, Myc A, Beals J, Thomas TP, Bander NH, Baker JR (2004). Synthesis and *in vitro* testing of J591 antibody-dendrimer conjugates for targeted prostate cancer therapy. Bioconju Chem.

[25] Bhadra D, Bhadra S, Jain NK (2005). PEGylated lysine based copolymeric dendritic micelles for solubilization and delivery of artemether. J Pharm Pharmaceut Sci.

[26] Wiwattanapatapee R, Lomlim L, Saramunee K (2003). Dendrimers conjugates for colonic delivery of 5-aminosalicylic acid. J Control Release.

[27] Tomalia DA, Baker H, Dewald J, Hall M, Kallos G, Martin S (1985). A new class of polymers: starburst-dendritic macromolecules. Polymer J.

[28] Eichman JD, Bielinska AU, Kukowska-Latallo JF, Baker JR (2000). The use of PAMAM dendrimers in the efficient transfer of genetic material into cells. Pharm Sci Technol Today.

[29] Kojima C, Kono K, Maruyama K, Takagishi T (2000). Synthesis of Poly amidoamine dendrimers having poly(ethylene glycol) grafts and their ability to encapsulate anticancer drugs. Bioconj Chem.

[30] Asthana A, Chauhan AS, Diwan PV, Jain NK (2005). Poly(amidoamine) (PAMAM) dendritic nanostructures for controlled site-specific delivery of acidic anti-inflammatory active ingredient. AAPS Pharm Sci Tech.

[31] Bryant LH, Brechbiel MW, Wu C, Bulte JW, Herynek V, Frank JA (1999). Synthesis and relaxometry of high-generation (G 5.5, 7, 9 and 10) PAMAM dendrimer-DOTA-gadolinium chelates. J Magn Reson Imaging.

[32] Wiwattanapatapee R, Carreno-Gomez B, Malik N, Duncan R (2000). Anionic PAMAM Dendrimers Rapidly Cross Adult Rat Intestine *In Vitro*: A Potential Oral Delivery System. Pharm Res.

[33] Jevprasesphant R, Penny J, Attwood D, D’Emanuele A (2004). Transport of dendrimer nanocarriers through epithelial cells via the transcellular route. J Control Release.

[34] Hong S, Bielinska AU, Mecke A, Keszler B, Beals JL, Shi X (2004). Interaction of Poly(amidoamine) Dendrimers with Supported Lipid Bilayers and Cells: Hole Formation and the Relation to Transport. Bioconjugate Chem.

[35] Tajarobi F, El-Sayed M, Rege BD, Polli JE, Ghandehari H (2001). Transport of poly amidoamine dendrimers across Madin–Darby canine kidney cells. Int J Pharm.

[36] El-Sayed M, Ginski M, Rhodes C, Ghandehari H (2002). Transepithelial transport of poly(amidoamine) dendrimers across Caco-2 cell monolayers. J Control Release.

[37] El-Sayed M, Rhodes CA, Ginski M, Ghandehari H (2003). Transport mechanism(s) of poly (amidoamine) dendrimers across Caco-2 cell monolayers. Int J Pharm.

[38] Wang Z, Itoh Y, Hosaka Y, Kobayashi I, Nokano Y, Maeda I (2003). Novel transdermal drug delivery system with Polyhydroxyalkanoate and starburst polyamidoamine dendrimer. J Biosci Bioeng.

[39] Wang Z, Itoh Y, Hosaka Y, Kobayashi I, Nokano Y, Maeda I (2003). Mechanism of enhancement effect of dendrimer on transdermal drug permeation through Polyhydroxyalkanoate matrix. J Biosci Bioeng.

[40] Chauhan AS, Sridevi S, Chalasani KB, Jain AK, Jain SK, Jain NK (2003). Dendrimer-mediated transdermal delivery: Enhanced bioavailability of Indomethacin. J Control Release.

[41] Yiyun C, Na M, Tongwen X, Rongqiang F, Xueyuan W, Xiaomin W (2006). Transdermal delivery of non-steroidal anti-inflammatory drugs mediated by polyamidoamine (PAMAM) dendrimers. J Pharm Sci.

[42] D’Emanuele A, Jevprasesphant R, Penny J, Attwood D (2004). The use of a dendrimer-propranolol prodrug to bypass efflux transporters and enhance oral bioavailability. J Control Release.

[43] Florence AT, Sakthivel T, Toth I (2000). Oral uptake and translocation of a polylysine dendrimer with a lipid surface. J Control Release.

[44] Sakthivel T, Toth I, Florence AT (1999). Distribution of a lipidic 2.5 nm diameter dendrimer carrier after oral administration. Int J Pharm.

[45] Wiwattanapatapee R, Lomlim L, Saramunee K (2003). Dendrimers conjugates for colonic delivery of 5-aminosalicylic acid. J Control Release.

[46] Na M, Yiyun C, Tongwen X, Yang D, Xiaomin W, Zhenwei L (2006). Dendrimers as potential drug carriers, Part II: Prolonged delivery of ketoprofen by *in vitro* and *in vivo* studies. Eur J Med Chem.

[47] Tomalia DA, Naylor AM, Goddard WA (1990). Starburst dendrimers, molecular-level of size, shape, surface chemistry, morphology and flexibility from atoms tomacroscopic matter. Angew Chem Int Ed Engl.

[48] Jevprasesphant R, Penny J, Attwood D, McKeown NB, D’Emanuele A (2003). Engineering of Dendrimer Surfaces to Enhance Transepithelial Transport and Reduce Cytotoxicity. Pharm Res.

